# Sub-acute and long-term effects of ayahuasca on affect and cognitive thinking style and their association with ego dissolution

**DOI:** 10.1007/s00213-018-4988-3

**Published:** 2018-08-13

**Authors:** M. V. Uthaug, K. van Oorsouw, K. P. C. Kuypers, M. van Boxtel, N. J. Broers, N. L. Mason, S. W. Toennes, J. Riba, J. G. Ramaekers

**Affiliations:** 1 Department of Neuropsychology and Psychopharmacology, Faculty of Psychology and Neuroscience, Maastricht University, Maastricht, The Netherlands; 2 Department of Clinical Sciences, Faculty of Psychology and Neuroscience, Maastricht University, Maastricht, The Netherlands; 3 Department of Psychiatry and Psychology, Faculty of Health, Medicine and Life Sciences, Maastricht University, Maastricht, The Netherlands; 4 Department of Methodology and Statistics, Faculty of Psychology and Neuroscience, Maastricht University, Maastricht, The Netherlands; 5 Institute of Legal Medicine, Goethe University of Frankfurt, Frankfurt, Germany

**Keywords:** Ayahuasca, Field study, Creative thinking, Affect, Long-term effects, Mindfulness

## Abstract

**Rationale:**

Ayahuasca is a psychotropic plant tea from South America used for religious purposes by indigenous people of the Amazon*.* Increasing evidence indicates that ayahuasca may have therapeutic potential in the treatment of mental health disorders and can enhance mindfulness-related capacities. Most research so far has focused on acute and sub-acute effects of ayahuasca on mental health-related parameters and less on long-term effects.

**Objectives:**

The present study aimed to assess sub-acute and long-term effects of ayahuasca on well-being and cognitive thinking style. The second objective was to assess whether sub-acute and long-term effects of ayahuasca depend on the degree of ego dissolution that was experienced after consumption of ayahuasca.

**Results:**

Ayahuasca ceremony attendants (*N* = 57) in the Netherlands and Colombia were assessed before, the day after, and 4 weeks following the ritual. Relative to baseline, ratings of depression and stress significantly decreased after the ayahuasca ceremony and these changes persisted for 4 weeks. Likewise, convergent thinking improved post-ayahuasca ceremony up until the 4 weeks follow-up. Satisfaction with life and several aspects of mindfulness increased the day after the ceremony, but these changes failed to reach significance 4 weeks after. Changes in affect, satisfaction with life, and mindfulness were significantly correlated to the level of ego dissolution experienced during the ayahuasca ceremony and were unrelated to previous experience with ayahuasca.

**Conclusion:**

It is concluded that ayahuasca produces sub-acute and long-term improvements in affect and cognitive thinking style in non-pathological users. These data highlight the therapeutic potential of ayahuasca in the treatment of mental health disorders, such as depression.

## Introduction

Ayahuasca is a psychotropic plant tea from South America used for healing purposes by indigenous people of the Amazon (Schmid 2012). It is known under various names like *yage, caapi, natem, mihi, dapa, daime*, or *hoasca*. Ayahuasca is prepared from the *Psychotria viridis* bush that contains the serotonergic 2A receptor agonist *N*,*N*-dimethyltryptamine (DMT) and the *Banisteriopsis caapi* liana that contains β-carboline alkaloids such as harmine, harmaline, and tetrahydroharmine (Palhano-Fontes et al. 2015; Riba et al. 2001). The β-carboline alkaloids inhibit the action of monoamine oxidase (MAO) that is responsible for breaking down DMT (Godinho et al. 2017). The inhibition of MAO allows DMT to reach the central nervous system for a prolonged period of time, causing intense alterations in sensory integration and awareness (Riba et al. 2001). The psychotropic effects of ayahuasca correspond with those induced by other psychedelics such as LSD, mescaline, and psilocybin (McKenna 2004). Psychotropic effects of ayahuasca start between 30 and 60 min post-administration, reaching maximum intensity between 60 to 120 min, and can last up to 4 h after administration (Riba et al. 2001).

The acclaimed benefits of ayahuasca are both spiritual and psychotherapeutic (Trichter 2010) and may be due to a symbiotic action between pharmacology and an altered state of consciousness (Re et al. 2016). Interviews with healthy ayahuasca users suggest that ayahuasca elicits spiritual insights, changed worldviews, and a new, more positive orientation towards life (Bouso et al. 2012; Grob et al. 1996; Halpern et al. 2008). Adolescents who regularly consume ayahuasca show less signs of anxiety and are more optimistic, self-confident, insistent, and emotionally mature than their peers (Da Silveira et al. 2005). Taken together, evidence suggests that ayahuasca may be a useful additive to psychotherapy to promote personal reflection and insights about attitude and belief (Trichter et al. 2009). The efficacy of this approach, however, depends on how well insights gained during the ayahuasca experience are integrated into everyday life (Frecska et al. 2016).

There is also evidence that ayahuasca affects thinking style. For example, the acute intake of ayahuasca led to significant increase in two facets of the Five Facets Mindfulness Questionnaire, i.e., non-judgment and non-reactivity (Soler et al. 2016). This suggests that ayahuasca may foster acceptance of thoughts and feelings experienced by the individual, which may be therapeutic for individuals who experience persistent negative thoughts. Increased acceptance of thoughts and feelings might have therapeutic benefits for patients with for example depression by allowing them to not judge or react to for example rumination. Furthermore, it has been demonstrated that ayahuasca acutely increases creative, divergent thinking while decreasing convergent thinking (Kuypers et al. 2016). The former represents a style of thinking whereby new ideas can be generated in a context in which more than one solution is correct, whereas the latter refers to a style of thinking where there is only one optimal solution to a problem. Interestingly, changes in divergent thinking help to strengthen psychological flexibility and allow adaptive coping styles (Forgeard and Elstein 2014). Imaging studies have shown that enhanced mindfulness capabilities are associated with increased functional brain connectivity (Sampedro et al. 2017;Viol et al. 2017).

Most research on ayahuasca has focused on acute and sub-acute effects on subjective well-being. However, there are also indications that effects of ayahuasca last far beyond the (sub) acute phase. Previous research demonstrated that a single oral dose of ayahuasca decreased depressive symptoms in three females as measured by the Hamilton Rating Scale for Depression Scale (HAM-D) (De Lima Osório et al. 2011). Depressive symptoms decreased by 79% on day 1 after intake and remained at 66% below their baseline 2 weeks after intake. More recently, 17 patients who received an oral dose of ayahuasca (2.2 mL/kg) showed a significant reduction in depressive symptoms within a day following treatment which was still present 21 days later (Sanches et al. 2016). This suggests that ayahuasca has fast-acting antidepressant properties that can last for up to 3 weeks. It has also been suggested that long-term therapeutic impact of psychedelics in part depends on the quality of psychedelic experience such as the occurrence of a profound psychological insight or the experience of ego dissolution (Roseman et al. 2017).

The present study aimed to assess whether administration of ayahuasca produces long-lasting changes on affect and creativity. The second objective was to assess whether the acute and long-term effects of ayahuasca depend on the degree of ego dissolution that was experienced during the ceremony. In the current study, we predicted that participants’ self-reported mindfulness and life satisfaction and creative divergent thinking would increase after the ayahuasca ceremony as compared to baseline. Moreover, we expected that symptoms of depression, anxiety, and stress-related symptoms would decrease after ayahuasca intake as compared to baseline. We furthermore expected that ayahuasca-induced changes would still be present 4 weeks after the ceremony as compared to baseline. Finally, we expected that positive changes in the dependent variables would be correlated with subjective ratings of ego dissolution.

## Methods

### Participants

Data were collected at ayahuasca ceremonies at different locations in Colombia (*N* = 27) and the Netherlands (*N* = 30). On site, participants of the ayahuasca ceremony were invited to enter the study. Exclusion criteria included non-fluency in Spanish, Dutch, or English.

A total of 57 participants from either location consented after goals and methods of the study were explained. This study was approved by the standing ethical review committee. Participation was voluntary and no incentives to participate were provided. The participants were not affiliated to any ayahuasca religion (e.g., União do Vegetal, or Santo Daime).

#### Group 1: the Dutch sample

Thirty volunteers were included at several ayahuasca ceremonies in the Netherlands (40% males and 60% females). Most participants were from Europe (93.3%) while the rest of the participants were from Asia (3.3%) and North America (3.3%). Their motivation for ayahuasca use included “understanding myself” (73.3%), “solving issues” (20%), and “curiosity” (6.7%). The highest completed level of education was higher vocational education (70%), high school (16.7%), a scientific education (10%), or elementary school (3.3%). None of the participants were currently on any medication that could have affected their ayahuasca intake. In total, 13 participants reported they had no previous experience with ayahuasca (43.3%). Twenty participants (66.7%) reported having had experience with other entheogenic drugs in the past, whereas 10 participants (33.3%) reported they had no experience with other entheogenic drugs. Finally, 23 participants reported they were not diagnosed with any mental health disorder (76.7%), 3 participants reported they suffered from depression (10%), 1 participant reported anxiety (3.3%), 2 participants reported personality disorders (6.6%), and 1 participant reported to suffer from addiction (3.3%).

#### Group 2: the Colombian sample

Twenty-seven volunteers (33.3% males, 66.7% females) from several locations in Colombia (Bogota, Bucaramanga, and Cali) completed the test battery. Most participants were from South America (70.4%) while the rest of participants were from Africa (11.1%) and North America (18.5%). Their reported motivation for ayahuasca use included understanding myself (40.7%), solving issues (18.5%), curiosity (3.7%), and other (37.0%). Most of the participants held a scientific degree (88.9%), while others held a high school degree (11.1%). None of the participants were currently on any medication that could affect their ayahuasca intake. In total, 11 participants reported they had no experience with ayahuasca (40.7%). Nineteen participants (70.4%) reported having had experience with other entheogenic drugs in the past. Regarding mental health disorders, 15 participants reported they were not diagnosed with any mental health disorder (55.6%), 4 participants reported they suffered from depression (14.8%), 2 participants suffered from anxiety (7.4%), and the rest of the participants did not answer this question.

### Study procedure

In this observational study, assessments were taken during three separate sessions: at baseline, the day after, and 4 weeks after the ayahuasca ceremony. In total, 57 participants completed the session pre- and post-ayahuasca, and 31 participants completed the 4-week follow-up. Attendants of the ceremony received an introduction of the research aims and were asked whether they would like to participate and signed the informed consent. They completed a 30-min test battery consisting of questionnaires and a psychometric test at baseline prior to the ceremony. All ayahuasca ceremonies were conducted during the night. The test battery was repeated once again on-site when the participants woke up the next day. In addition, participants also completed an ego dissolution questionnaire. The follow-up ayahuasca assessment at 4 weeks after the ceremony was administered through an online survey created in Qualtrics.

### Setting

#### Group 1: the Dutch sample

The ayahuasca ceremonies in the Netherlands were held in a tipi outside or in a big hotel room. The goal of the ceremony was to relieve psychological or physical issues, increase well-being, or to gain personal insights. In each ceremony, there were at least two or more experienced ayahuasca facilitators with a background in healing and/or coaching. Personal intakes were done by a naturopathic doctor that screened participants for their motivation, medicine use, and medical and psychological history. Participants on antidepressant medication or with an anxiety disorder were not allowed to participate.

#### Group 2: the Colombian sample

The ayahuasca brew in Colombia was taken in a secluded location in the rainforest on top of a mountain or in the courtyard of an eco-village. Participants arrived at the location in the evening and remained in a dimly-lit ceremonial building called *maloca*, used for ayahuaca ceremonies. The ceremonies were primarily arranged to facilitate personal healing and development while also being part of the culture in South America. The shamans would provide support for participants if they were going through a challenging time during the ceremony. The shaman screened participants prior to the ceremony and would not allow participants on antidepressants or those with a mental health disorder to participate.

In both countries, the ceremonies were held by experienced shamans with a background in healing with plant medicines, trained in the Amazon during the ceremony. The shaman and the organizers would sing *icaros* (this is the term that is commonly used to describe the medicine songs performed in these ceremonies) and play instruments (guitar, drums, flute). Furthermore, the neuropathic doctor in Netherlands and the shaman in Colombia both provided participants with preparatory instructions (e.g., dietary preparation). Dietary preparation included = not eating (red) meat and foods containing salt, sugar, or fats reduce tyramine levels in the body. Lower tyramine levels reduce side effects like increased cardiovascular activity, nausea, and headaches that can be caused by the MAO-Inhibitor in the brew. Additional advice was not to subject oneself to stress (e.g., watching violent TV programs, news), and sexual intercourse to conserve energy, calm the mind, and to increase mindful introspection, which, according to Amazonian tradition, improves the healing effects of the plant medicine and will reduce purging and negative side effects of the medicine.

### Ayahuasca samples

In total, 4 ayahuasca samples were obtained (two from Colombia and two from The Netherlands). The alkaloid concentrations of the ayahuasca preparations were determined after dilution using high-performance liquid chromatography-electrospray ionization-time-of-flight mass spectrometry (HPLC-TOF MS) which was calibrated with pure reference substances of *N*,*N*-dimethyltryptamine (DMT; Cerilliant, Round Rock TX, US), harmine and harmaline (Aldrich Chemistry, St. Louis MO, US).

From the alkaloid concentrations, it was determined that a 200-ml portion of Netherlands 1 contained 371.6 mg of DMT, 485.5 mg of harmine, and 892 mg of harmaline; a 200-ml portion of Netherlands 2 contained 915.4 mg of DMT, 971.8 mg of harmine, and 38.1 mg; a 200 ml portion of Colombia 1 contained 189.4 mg of DMT, 1261.7 mg of harmine, and 69.8 mg of harmaline; and a 200 ml portion of Colombia 2 contained 500.5 mg of DMT, 827.4 mg of harmine, and 57.4 mg of harmaline.

## Measures

### Picture concept task

A creativity task with non-verbal stimuli was used, i.e., the picture concept task (PCT) (Kuypers et al. 2016). The PCT was composed of stimuli from the Wechsler Preschool and Primary Scale of Intelligence and the Wechsler Intelligence Scale for Children. Each stimulus contains between 4 and 12 color pictures shown in two or three rows. The participants are instructed to find an association between one of the pictures of each row. They are asked to provide the correct solution as there is only one correct answer. The correct answers are taken as the dependent measure of convergent thinking. To assess divergent thinking, participants were asked to provide as many alternative associations as possible by sticking to the rule; one item per row. This is the regular instruction included in measures of divergent thinking, and it is used to calculate several parameters, i.e., originality, fluency, and the ratio of both, which reflect quantity and quality of divergent thinking. Fluency is defined as the number of alternative associations. Originality is calculated by evaluating the originality of the alternative association relative to those provided by all other participants in a session. Alternative answers that were uniquely reported by a single participant received an originality score of 2. Answers that were shared with a single participant were valued as 1, and answers that were shared by three or more participants were rated as zero. Mean originality (creativity) scores and ratio originality scores, weighed for fluency (originality/fluency), were used as measures of divergent thinking. Three parallel versions of the PCT were used at baseline and the two follow-up measures after the ceremony to avoid learning effects. Each parallel version consisting of 17 stimuli were shown, and participants had 30 s per stimulus.

### Subjective measures

The test battery in addition included four questionnaires, the Depression, Anxiety, and Stress Scale-21 (DASS-21), the Satisfaction with Life Scale (SWLS), the Five Facets Mindfulness Questionnaire (FFMQ), and the Ego Dissolution Inventory (EDI).

#### Depression, Anxiety, and Stress Scale

The Depression, Anxiety, and Stress Scale (DASS-21) is a shorter version of the originally 42-item long self-report questionnaire (Henry and Crawford 2005). Sample items for each subscale include the following; “I couldn’t seem to experience any positive feeling at all,” “I was aware of dryness of my mouth,” and “I found it hard to wind down,” respectively. The purpose of this scale is to measure constructs of depression, anxiety, and stress. The participants responded by rating the concordance with each statement from 0 (did not apply to me at all) to 3 (applied to me very much, or most of the time). The subscale scores for depression, anxiety, and stress are calculated by summing the scores for the relevant items, multiplied by 2, given that the original DASS has 42 questions. In this study, the English version of the scale was used, in addition to a validated version translated into Spanish and Dutch. The total scale of the English DASS-21 had a coefficient alpha of 0.93. Subscale coefficient alphas were also high (α depression = 0.88; α anxiety = 0.82; α stress = 0.90). The total scale of the Spanish version of the DASS-21 had a Cronbach alpha coefficient of 0.96. Cronbach alpha of the Dutch version was 0.95 (Daza et al. 2002; Wardenaar et al. 2018).

#### Satisfaction with Life Scale

The Satisfaction with Life Scale (SWLS) is a 5-item self-report scale (Diener et al. 1985). Sample items include “In most ways my life is close to my ideal” and “So far I have gotten the important things I want in life.” The purpose of the scale is to assess someone’s satisfaction with life. The items are answered on a Likert-scale ranging from 1 “Strongly disagree” to 7 “Strongly agree.” The total score is obtained by summarizing the ratings from each item and ranges between 5 and 35, with higher scores indicating a greater life satisfaction. The scale has good psychometric properties. The original SWLS in English has a Cronbach’s alpha of 0.87 and was used in addition to a validated translation in Spanish with a Cronbach’s alpha coefficient of 0.88 and a Dutch translation with a Cronbach’s alpha coefficient of 0.85 (Beuningen 2012; Vazquez et al. 2013).

#### Five Facets Mindfulness Questionnaire

The Five Facets Mindfulness Questionnaire (FFMQ) is a 39-item self-report questionnaire (Baer et al. 2006) assessing five different factors: (1) observe: noticing experience that are both internal and external such as for example thoughts and emotions, e.g., “When I’m walking, I deliberately notice the sensations of my body moving”; (2) describe: describing internal experiences, e.g., “I’m good at finding words to describe my feelings”; (3) acting with awareness: focus on the present activity, e.g., “When I do things, my mind wanders off and I’m easily distracted”; (4) non-judgment: not evaluating the present experience, e.g., “I criticize myself for having irrational or inappropriate emotions”; (5) non-reaction: allowing thoughts and feelings to come without acting or reacting upon them, e.g., “I perceive my feelings and emotions without having to react to them”. The purpose of this scale is to obtain an understanding of an individual’s mindfulness-related capacities. The participants answered the FFMQ by rating the concordance with each statement on a 5-point Likert-scale that ranges from 1 (never true) to 5 (very often or always true). The total FFMQ score is obtained by adding the subscale scores. The scale has shown good internal consistency. Cronbach’s alpha was α non-reaction = 0.75, α non-judgment = 0.87, α describe = 0.91, α observe = 0.83, and α awareness = 0.87. In this study, the original English version of the FFMQ was used in addition to a validated translation in Spanish and Dutch. The Cronbach’s alpha for the Spanish translation ranges from 0.80 to 0.91 (Cebolla et al. 2012). Cronbach’s alpha for the Dutch translation ranges from 0.74 to 0.90 (Veehof et al. 2011).

#### Ego Dissolution Inventory

The Ego Dissolution Inventory (EDI) is an eight-item self-report scale that assesses the participant’s experience of ego dissolution (Nour et al. 2016). Sample items for the scale includes the following: “I experienced a dissolution of my ‘self’ or ego” and “I felt at one with the universe.” The purpose of this scale is to acquire a better understanding of the experiences the participants had about ego dissolution during the ayahuasca ceremony. The participants answered the scale with endpoints of either 0 = “No, not more than usually” or 100 = “Yes I experience this completely/entirely.” The EDI is scored by calculating the mean of all the 8 items (range 0–100). The higher the total score, the stronger the experience of ego dissolution. In this study, the original English version of the EDI was used in addition to a non-validated Spanish and Dutch version.

### Statistical analyses

Data was analyzed with the Statistical Package for the Social Sciences (SPSS). We carried out linear mixed model analysis that included session (three levels: baseline, 1 day after ayahuasca and 4 weeks after ayahuasca) as the within-subject factor, country (two levels: Colombia and the Netherlands) as between-group factor, and the session × country interaction. The covariance structure was chosen according to best fit and could vary across outcome variables. Different covariance structures used included compound symmetry heterogeneous (CSH) as well as first lag autoregressive (AR1) structures. Significant main effects of session and session × country were followed by separate contrasts between baseline and follow-up session with Bonferroni adjustments for multiple comparisons. For outcome parameters showing a significant main effect of session, a second mixed model analysis was conducted with session (two levels, baseline and the day after) and experience with ayahuasca (two levels, yes or no) as main factors, to determine whether the sub-acute response to the ayahuasca ceremony differed between participants who experienced ayahuasca before or who had no previous experience. Pearson’s correlations were carried out to investigate the association between the level of ego dissolution during the ayahuasca ceremony and changes in outcome measures over time relative to baseline.

## Results

Overall, 57 participants completed parts of the test battery at baseline and during the day after the ceremony. A total of 31 participants also completed parts of the online test battery at 4 weeks after the ayahuasca ceremony.

### Picture concept test

**Fig. 1:**
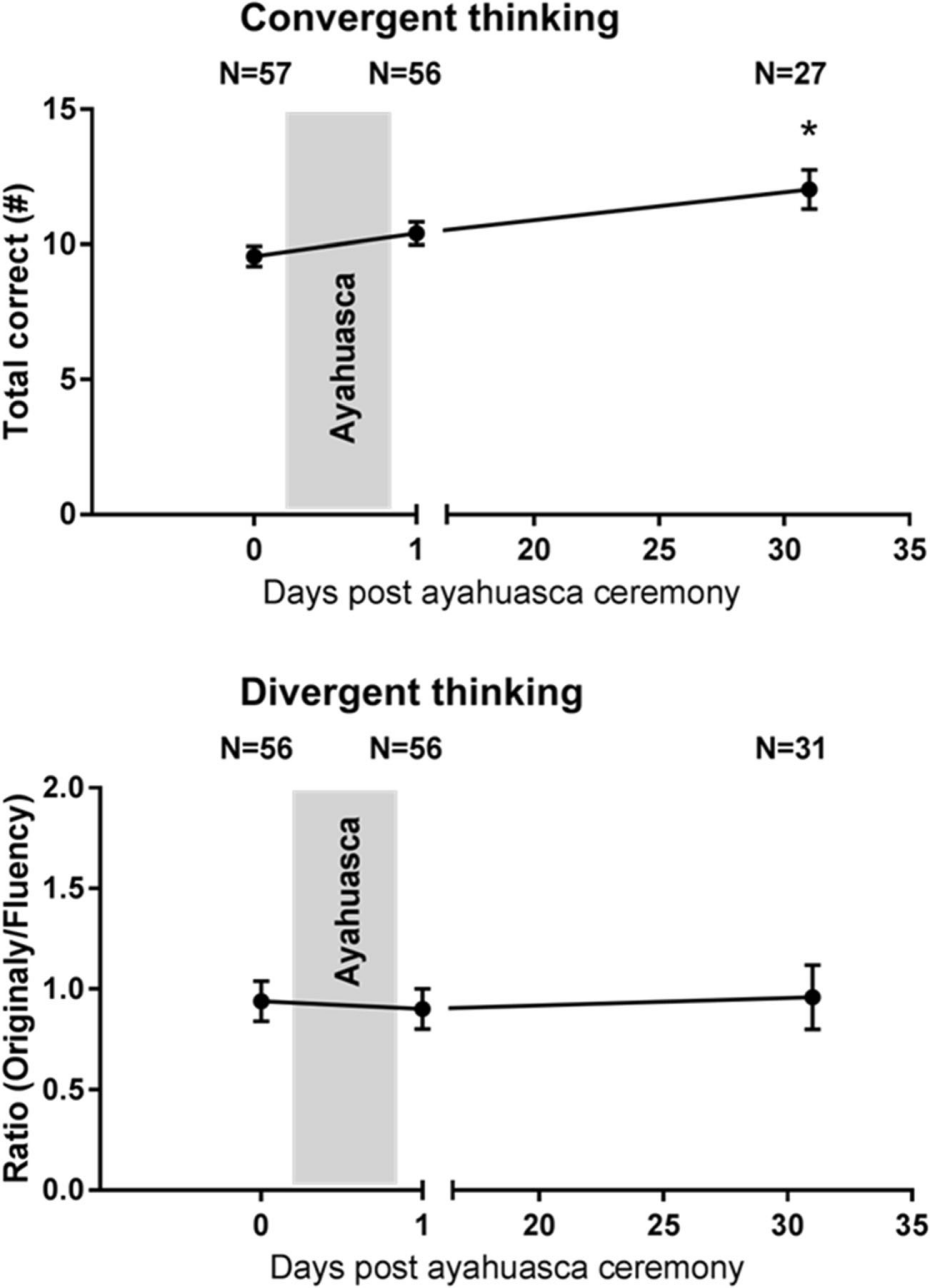
Mean (SE) total correct solutions (convergent thinking) and the ratio between originality and fluency (divergent thinking) as a function of time after ayahuasca (*p < .05)

**Fig. 2:**
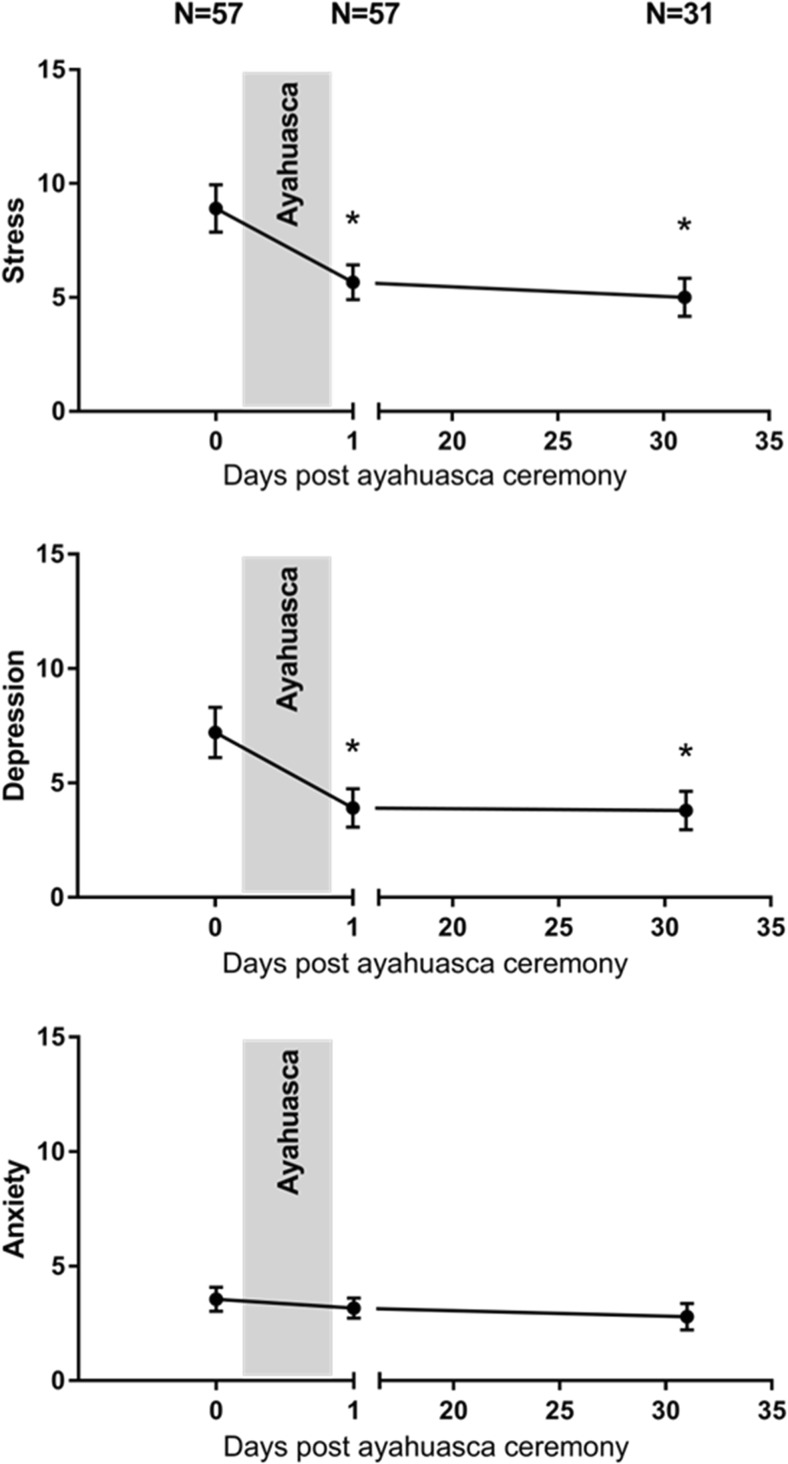
Mean (SE) subjective ratings of stress, depression, and anxiety as a function of time after ayahuasca (*p < .05)

**Fig. 3:**
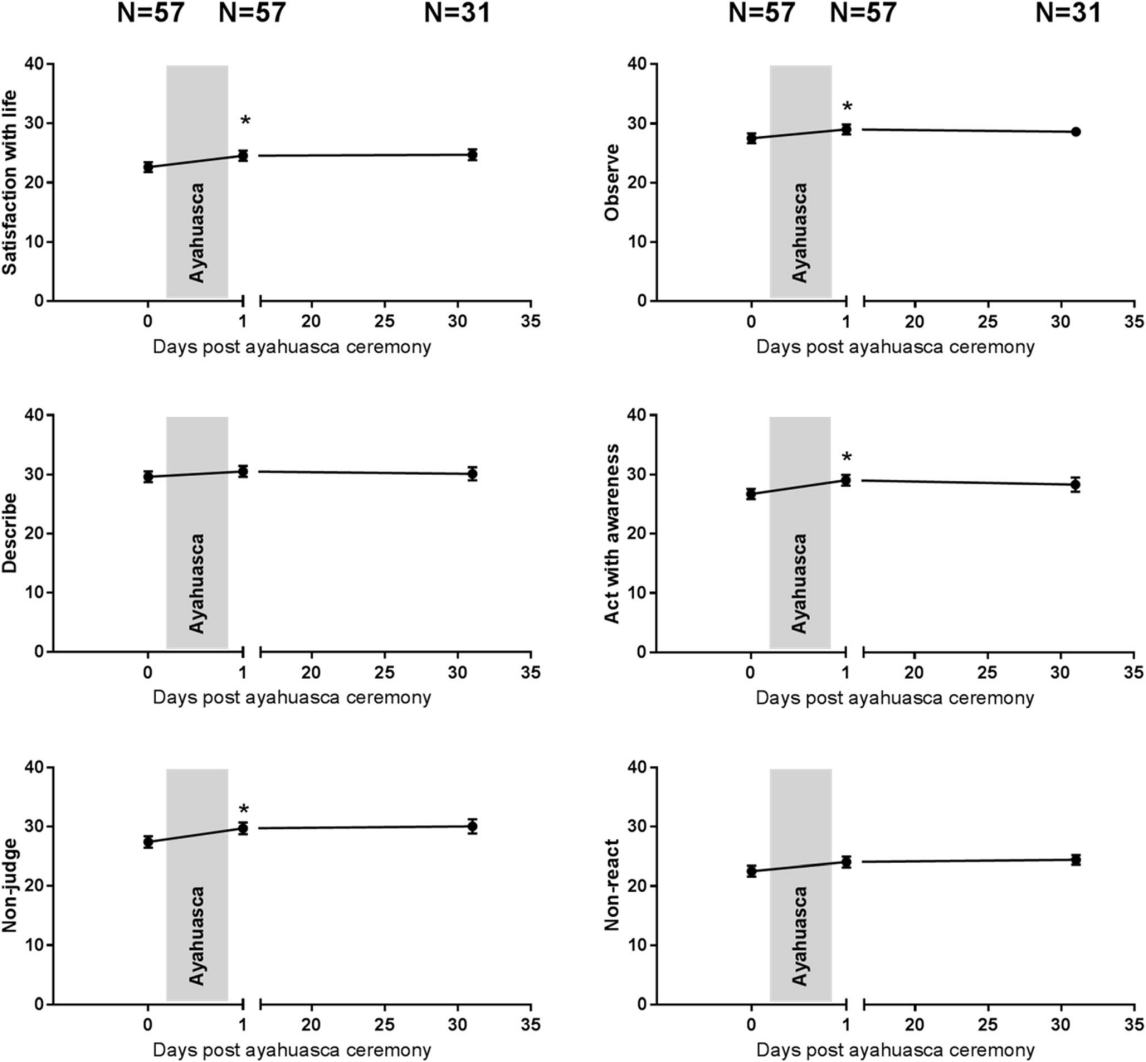
Mean (SE) subjective ratings of satisfaction with life and five dimensions of mindfulness as assessed with the FFMQ as a function of time after ayahuasca (*p < .05)

The mixed-model analysis revealed a significant main effect of session (F_2, 43.8_ = 7.06; *p* = .002) on convergent thinking. Separate contrasts revealed that the number of correct solutions increased after the ayahuasca ceremony, as compared to baseline. The increment in convergent thinking on the day after the ceremony approached significance (*p* = .09) and reached full significance 4 weeks after (*p* = .003). Convergent thinking was not affected by country or session × country. None of the divergent thinking parameters were affected by any of the main factors or their interaction. Mean (SE) performance on the convergent correct and divergent assignment as a function of time after ayahuasca is shown in Figs. 1, 2, and 3.

### Subjective measures

#### Depression, Anxiety, and Stress Scale

Mixed-model analysis revealed main effects of session on stress (F_2, 72.6_ = 7.3; *p* < .001) and depression (F_2, 78.9_ = 7.1; *p* = .001). Separate contrast revealed that subjective ratings of stress and depression decreased on the day after the ayahuasca ceremony (*p* = .006 and *p* < .001, respectively) and as well at 4 weeks after (*p* = .002 and *p* = .013, respectively). There was also a main effect of country indicating lower stress (F_1, 52.3_ = 17.3; *p* < .001) and depression (F_1, 46_ = 16.4; *p* < .001) scores in the Colombian sample as compared to the Dutch sample. The interaction between session and country did not reach significance for stress and depression score. Anxiety ratings were not affected by any of the main factors.

#### Satisfaction with Life Scale

Mixed-model analyses revealed a main effect of session (F_2, 46.4_ = 3.7; *p* = .031). Contrasts showed that satisfaction with life significantly increased the day after the ayahuasca ceremony (*p* = 0.027) but not 4 weeks later, as compared to baseline. Satisfaction with life ratings were significantly higher in the Colombian sample as compared to the Dutch sample (F_1, 51.6_ = 20.4; *p* = *p* < .001) There was no interaction between session and country.

#### Five Facets Mindfulness Questionnaire

Main effects of session reached significance on four mindfulness parameters, i.e., observe (F_2, 69.4_ = 4.3; *p* = .018), non-judge (F_2, 68.4_ = 4.4; *p* = .016), aware (F_2, 60.4_ = 5.5; *p* = .006), and non-reaction (F_2, 75.7_ = 3.4; *p* = .038). Describe was not affected by session. Separate contrasts revealed improvements in observe (*p* = 0.15), non-judge (*p* = .012), and aware (*p* = .004) on the day after the ayahuasca ceremony as compared to baseline. Comparisons between FFMQ measures after 4 weeks and baseline revealed no significant differences. Ratings of aware were significantly higher in the Colombian sample as compared to the Dutch sample (F_1, 57.7_ = 8.6; *p* = .005). There were no differences between countries for other FFMQ parameters, and interactions between session and country never reached significance.

#### Ego Dissolution Inventory

**Fig. 4:**
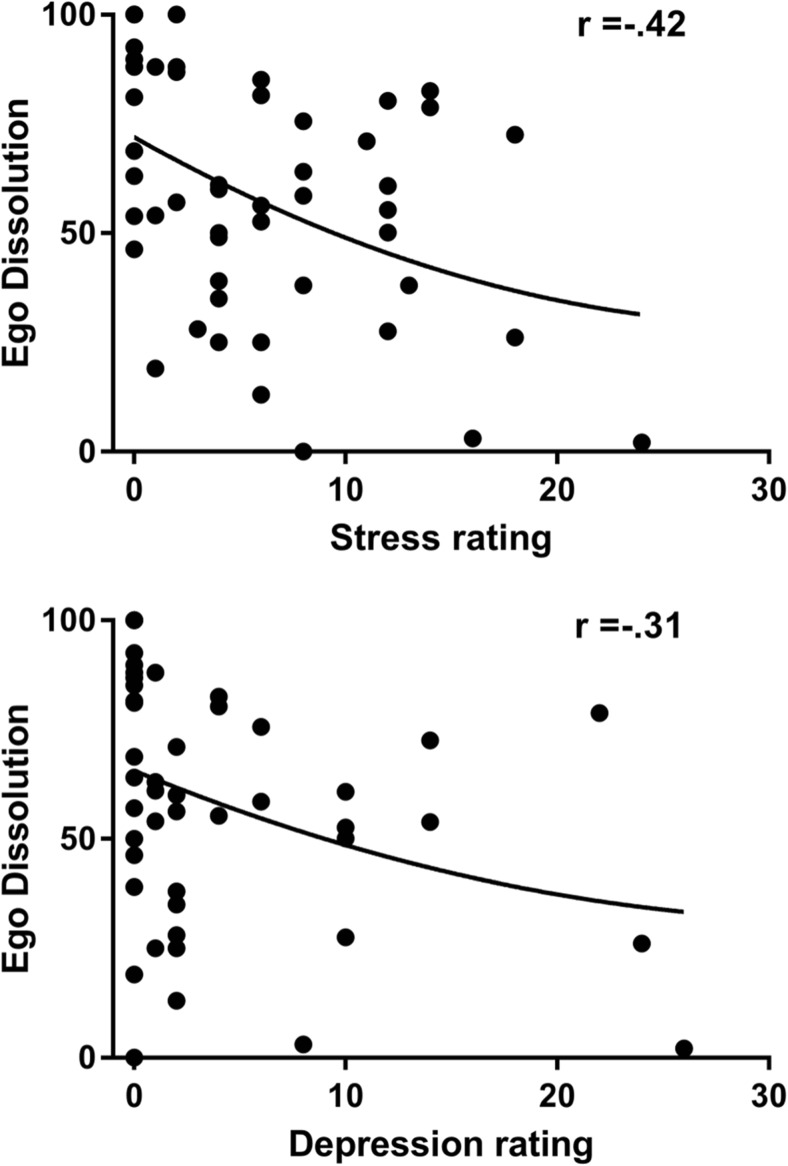
Pearson correlation between ego dissolution and subjective stress (upper panel) and between ego dissolution and depression ratings (lower panel) on the day after the ayahuasca ceremony

Mean (SD) ego dissolution rating was 60 (27.3). Overall, total EDI rating varied between 0 (no dissolution) and 100 (maximal dissolution). On the day following the ayahuasca ceremony, EDI scores were negatively correlated to DASS ratings of stress (*r* = − .42; *p* = .007) and depression (*r* = − .31; *p* < .001) and positively correlated to FFMQ ratings of awareness (*r* = .39; *p* = .003), non-judgment (*r* = .36; *p* = .007), non-reactivity (*r* = .39; *p* = .003), and satisfaction with life (*r* = .37; *p* = .007). Subjective ratings at 4 weeks after the ceremony did not correlate with EDI, except for non-reactivity (*r* = .39; *p* = .028). Scatterplots showing the negative relation between EDI and ratings of stress and depression on day 1 are shown in Fig. 4. EDI did not correlate with the PCT parameter of convergent thinking.

### Experience with ayahuasca

The sub-acute effect of ayahuasca did not differ between participants with no (*N* = 24) or previous experience (*N* = 33) with ayahuasca for any of the above outcome parameters that were significantly affected by a main effect of session. There were no main effects of experience and session × experience.

## Discussion

The primary aim of the present study was to assess sub-acute and long-term impact of ayahuasca use on affect and cognitive thinking style. A total of 57 visitors of ayahuasca ceremonies in Colombia and the Netherlands consented to participate in this observational study and were asked to complete subjective questionnaires and a creative thinking task prior to an ayahuasca ceremony, the day after, and about 4 weeks later. Baseline and sub-acute assessments were conducted on-site whereas the 4-week follow-up was conducted online. The 4-week follow-up was completed by about 54% of the participants. Relative to baseline, ratings of depression and stress significantly decreased after the ayahuasca ceremony and these changes persisted for at least 4 weeks. Likewise, convergent thinking improved after the ayahuasca ceremony even at the 4-week follow-up. Satisfaction with life and several aspects of mindfulness increased the day after the ceremony, but these changes failed to reach significance 4 weeks after. Changes in affect, satisfaction with life, and mindfulness were significantly related to the level of ego dissolution experienced during the ayahuasca ceremony.

Subjective ratings of stress and depression significantly decreased by 36 and 46% respectively during the day after the ayahuasca ceremony. Ratings of stress and depression remained significantly lower throughout the following month, suggesting that a single ayahuasca ceremony can bring about changes in affect that last for a prolonged period of time. Antidepressant properties of ayahuasca are not unexpected given that ayahuasca brews contain β-carboline alkaloids that act as MAO-A inhibitors which are known for their antidepressant actions (Finberg and Rabey 2016). Sub-acute reductions in depressive symptoms have been reported previously in first time (Barbosa et al. 2005) and regular ayahuasca users (Bouso et al. 2012). A pilot, open-label trial in six patients suffering from recurrent or major depressive disorder however showed significant reductions of up to 82% in depressive scores between baseline and 1, 7, and 21 days after intake of a single ayahuasca dose (Osório et al. 2015). These data were recently replicated in a larger open-label trial that involved 17 depressed patients (Sanches et al. 2016). Together, these studies support the notion that single doses of ayahuasca can reduce symptomatology in depressed patients as well as in a broad spectrum of users who display non-pathological levels of depression and that such changes persist over a prolonged period of time.

The mechanism underlying the long-term effects of ayahuasca on affect is presently unknown. Animal studies suggest that *Banisteriopsis caapi* preparations produce antidepressant activity through hippocampal neurogenesis (Farzin and Mansouri 2006; Fortunato et al. 2010). A recent study demonstrated that harmine, tetrahydroharmine, and harmaline, the three main alkaloids present in the *Banisteriopsis caapi*, stimulate adult neurogenesis in vitro using hippocampal progenitor cells from adult mice (Morales-Garcia et al. 2017). Neurogenesis has also been suggested to underlie the acute and long-term antidepressant properties of other psychedelic compounds such as ketamine (Duman and Aghajanian 2012; Ma et al. 2017) and psilocybin (Catlow et al. 2013; Idell et al. 2017). Neurogenesis and synaptic plasticity may play a crucial role in how neural circuits regulate their excitability and connectivity and may link the neurobiology of depression to the therapeutic effects of glutamatergic drugs such as ketamine (Abdallah et al. 2015). In that respect, it is of interest that changes in glutamate transmission following ayahuasca ceremonies have been associated to improvements in certain aspect of mindfulness as well (Sampedro et al. 2017).

Ayahuasca also produced significant sub-acute improvements in subjective ratings of mindfulness and satisfaction with life. Subjects felt that they were more non-judgmental, acted with more awareness, and were more observant on the day following ayahuasca. Similar findings have been reported in an observational study that compared aspects of mindfulness before and 24 h after an ayahuasca ceremony using the same FFMQ questionnaire (Soler et al. 2016). In the present study, ratings of mindfulness and satisfaction with life increased by 5–8%, relative to baseline. On average, similar increments in mindfulness were obtained at 4 weeks after the ayahuasca ceremony, but this time, these no longer achieved statistical significance. It should be noted that ayahuasca-induced changes in mindfulness were much smaller than changes in symptoms of depression and stress. Consequently, the loss of statistical power caused by the drop in the response rate at the 4-week follow-up may have affected the probability of detecting changes as assessed with the FFMQ and SWL more than those assessed with the DASS, because changes in the former were small.

Exposure to the ayahuasca ceremony also increased convergent thinking as assessed with the picture concept task. The number of correctly detected associations increased with 9% sub-acutely and with 29% at the 4-week follow-up. At the latter time point, the increase in cognitive performance also reached statistical significance. The PCT task has previously been used in an observational ayahuasca study to show that the brew increases performance at the divergent, creative thinking assignment but decreases performance in convergent thinking assignments (Kuypers et al. 2016). Data from these studies however are not necessarily conflicting. In the latter study, PCT performance was assessed during the acute phase of the ayahuasca experience (i.e., 2 h after intake), whereas in the present study, PCT performance was assessed sub-acutely and a month after the ceremony. It is conceivable that divergent, flexible thinking will improve during the acute psychedelic, modified state of consciousness that ayahuasca produces but not thereafter. Convergent thinking may not prosper during a psychedelic experience but may improve afterwards because it relies on mindfulness capabilities such as acting with awareness that contribute to optimization of cognitive functioning (Lebudaa et al. 2015). Increments in convergent thinking may therefore coincide with improvements in mindfulness that were observed in the present sample of ayahuasca users.

It might be argued that post-ayahuasca changes in affect and thinking style that were observed in the present study are not related to a pharmacological effect of ayahuasca but due to uncontrolled confounders such as psychological expectations of participants prior to and after the ayahuasca ceremony. For example, participants may have been more stressed in anticipation of the ayahuasca ceremony which would explain why stress levels are higher at baseline, prior to the ceremony. Ideally, one would need to include a placebo group to control for the potential bias of expectation. This however is often impossible in observational studies that aim to assess the influence of ayahuasca in naturalistic environments. Yet, there are several strong indicators in the present study that suggest that changes in affect and thinking were directly related to the intake of ayahuasca. First, it was demonstrated that improvements in affect and cognition were significantly correlated with the strengths of ego dissolution during the ayahuasca experience. Higher ratings of ego dissolutions were associated with stronger reductions in symptoms of depression and stress and with improvements in mindfulness and satisfaction with life. This strongly suggests that changes in mood and thinking were intrinsically related to the actual ayahuasca experience. Second, anxiety levels were very low during baseline and follow-up sessions, which suggest that anxiety levels in anticipation of the ayahuasca ceremony were negligible. Finally, improvements in affect and cognition occurred independent of the previous ayahuasca experience of participants. The aforementioned rules out the possibility that changes were only apparent in first-time users who might be overly stressed by the novelty of the ayahuasca experience.

In sum, this study presents supporting evidence for sub-acute and long-term improvements in affect and cognitive thinking style in non-pathological participants of ayahuasca ceremonies. Moreover, it was shown that improvements in affect and mindfulness are larger in participants who experience strong levels of ego dissolution during the acute phase of ayahuasca exposure. These data highlight the therapeutic potential of ayahuasca in the treatment of mental health disorders, such as depression.

## References

[CR1] Abdallah CG, Salas R, Jackowski A, Baldwin P, Sato JR, Mathew SJ (2015). Hippocampal volume and the rapid antidepressant effect of ketamine. J Psychopharmacol.

[CR2] Baer RA, Smith GT, Hopkins J, Krietemeyer J, Toney L (2006). Using self-report assessment methods to explore facets of mindfulness. Assessment.

[CR3] Barbosa PCR, Giglio JS, Dalgalarrondo P (2005). Altered states of consciousness and short-term psychological after-effects induced by the first time ritual use of ayahuasca in an urban context in Brazil. J Psychoactive Drugs.

[CR4] Beuningen J (2012) The satisfactin with life scale examining construct validity. Statistics Netherlands 201209:3–23

[CR5] Bouso JC, González D, Fondevila S, Cutchet M, Fernández X, Barbosa PCR (2012). Personality, psychopathology, life attitudes and neuropsychological performance among ritual users of ayahuasca: a longitudinal study. PLoS One.

[CR6] Catlow BJ, Song S, Paredes DA, Kirstein CL, Sanchez-Ramos J (2013). Effects of psilocybin on hippocampal neurogenesis and extinction of trace fear conditioning. Exp Brain Res.

[CR7] Cebolla A, García-Palacios A, Soler J, Guillen V, Baños R, Botella C (2012). Psychometric properties of the Spanish validation of the five facets of mindfulness questionnaire (FFMQ). Eur J Psychiat.

[CR8] Da Silveira DX, Grob CS, de Rios MD, Lopez E, Alonso LK, Tacla C, Doering-Silveira E (2005). Ayahuasca in adolescence: a preliminary psychiatric assessment. J Psychoactive Drugs.

[CR9] Daza P, Novy DM, Stanley MA, Averill P (2002). The depression anxiety stress scale-21: Spanish translation and validation with a Hispanic sample. J Psychopathol Behav Assess.

[CR10] De Lima Osório F, De Macedo LRH, De Sousa JPM, Pinto JP, Quevedo J, de Souza Crippa JA, Hallak JEC (2011) The therapeutic potential of harmine and ayahuasca in depression: evidence from exploratory animal and human studies. The Ethnopharmacology of Ayahuasca: 75–86

[CR11] Diener E, Emmons RA, Larsen RJ, Griffin S (1985). The satisfaction with life scale. J Pers Assess.

[CR12] Duman RS, Aghajanian GK (2012). Synaptic dysfunction in depression: potential therapeutic targets. Science.

[CR13] Farzin D, Mansouri N (2006). Antidepressant-like effect of harmane and other beta-carbolines in the mouse forced swim test. Eur Neuropsychopharmacol.

[CR14] Finberg JP, Rabey JM (2016). Inhibitors of MAO-A and MAO-B in psychiatry and neurology. Front Pharmacol.

[CR15] Forgeard MJ, Elstein JG (2014). Advancing the clinical science of creativity. Front Psychol.

[CR16] Fortunato JJ, Reus GZ, Kirsch TR, Stringari RB, Fries GR, Kapczinski F, Hallak JE, Zuardi AW, Crippa JA, Quevedo J (2010). Effects of beta-carboline harmine on behavioral and physiological parameters observed in the chronic mild stress model: further evidence of antidepressant properties. Brain Res Bull.

[CR17] Frecska E, Bokor P, Winkelman M (2016). The therapeutic potentials of ayahuasca: possible effects against various diseases of civilization. Front Pharmacol.

[CR18] Godinho AF, Silva MC, Kawashima JD, Horta DF, Anselmo F, De Fraia D (2017) Ayahuasca modifies amphetamine self ingestion and modifies anxiety and locomotor activity in adolescent rats. Electronic J Biol 13:159–165

[CR19] Grob CS, McKenna DJ, Callaway JC, Brito GS, Neves ES, Oberlander G (1996). Human psychopharmacology of hoasca, a plant hallucinogen used in ritual context in Brazil. J Nerv Ment Dis.

[CR20] Halpern JH, Sherwood AR, Passie T, Blackwell KC, Ruttenber AJ (2008) Evidence of health and safety in American members of a religion who use a hallucinogenic sacrament. Med Sci Monit 14:15–22 18668010

[CR21] Henry JD, Crawford JR (2005). The short-form version of the depression anxiety stress scales (DASS-21): construct validity and normative data in a large non-clinical sample. Br J Clin Psychol.

[CR22] Idell RD, Florova G, Komissarov AA, Shetty S, Girard RB, Idell S (2017). The fibrinolytic system: a new target for treatment of depression with psychedelics. Med Hypotheses.

[CR23] Kuypers KP, Riba J, de la Fuente RM, Barker S, Theunissen EL, Ramaekers JG (2016). Ayahuasca enhances creative divergent thinking while decreasing conventional convergent thinking. Psychopharmacology.

[CR24] Lebudaa I, Zabelinab DL, Karwowskia M (2015). Mind full of ideas: a meta-analysis of the mindfulness–creativity link. Personal Individ Differ.

[CR25] Ma Z, Zang T, Birnbaum SG, Wang Z, Johnson JE, Zhang CL, Parada LF (2017) TrkB dependent adult hippocampal progenitor differentiation mediates sustained ketamine antidepressant response. Nature Communications 8:1668 10.1038/s41467-017-01709-8 PMC5698402 29162814

[CR26] McKenna DJ (2004). Clinical investigations of the therapeutic potential of ayahuasca: rationale and regulatory challenges. Pharmacol Ther.

[CR27] Morales-Garcia JA, Echeverry-Alzate V, Alonso-Gil S, Sanz-SanCristobal M, Lopez-Moreno JA, Gil C, Martinez A, Santos A, Perez-Castillo A (2017). Phosphodiesterase7 inhibition activates adult neurogenesis in hippocampus and subventricular zone in vitro and in vivo. Stem Cells.

[CR28] Nour MM, Evans L, Nutt D, Carhart-Harris RL (2016). Ego-dissolution and psychedelics: validation of the ego-dissolution inventory (EDI). Front Hum Neurosci.

[CR29] Osório FL, Sanches RF, Macedo LR, Santos RG, Maia-de-Oliveira JP, Wichert-Ana L, de Araujo DB, Riba J, Crippa JA, Hallak JE (2015). Antidepressant effects of a single dose of ayahuasca in patients with recurrent depression: a preliminary report. Rev Bras Psiquiatr.

[CR30] Palhano-Fontes F, Andrade KC, Tofoli LF, Santos AC, Crippa JA, Hallak JE, Ribeiro S, de Araujo DB (2015). The psychedelic state induced by ayahuasca modulates the activity and connectivity of the default mode network. PLoS One.

[CR31] Re T, Palma J, Martins JE, Simões M (2016). Transcultual perspective on consciousness: traditional use of ayahuasca in psychiatry in the 21st century in the western world. Cosmos and History.

[CR32] Riba J, Rodriguez-Fornells A, Urbano G, Morte A, Antonijoan R, Montero M, Callaway JC, Barbanoj MJ (2001). Subjective effects and tolerability of the south American psychoactive beverage Ayahuasca in healthy volunteers. Psychopharmacology.

[CR33] Roseman L, Nutt DJ, Carhart-Harris RL (2017). Quality of acute psychedelic experience predicts therapeutic efficacy of psilocybin for treatment-resistant depression. Front Pharmacol.

[CR34] Sampedro F, de la Fuente Revenga M, Valle M, Roberto N, Dominguez-Clave E, Elices M, Luna LE, Crippa JAS, Hallak JEC, de Araujo DB, Friedlander P, Barker SA, Alvarez E, Soler J, Pascual JC, Feilding A, Riba J (2017). Assessing the psychedelic “after-glow” in ayahuasca users: post-acute neurometabolic and functional connectivity changes are associated with enhanced mindfulness capacities. Int J Neuropsychopharmacol.

[CR35] Sanches RF, de Lima OF, Dos Santos RG, Macedo LR, Maia-de-Oliveira JP, Wichert-Ana L, de Araujo DB, Riba J, Crippa JA, Hallak JE (2016). Antidepressant effects of a single dose of ayahuasca in patients with recurrent depression: a SPECT study. J Clin Psychopharmacol.

[CR36] Schmid JT (2012). The myth of ayahuasca. Int J Religion Spiritual Soc.

[CR37] Soler J, Elices M, Franquesa A, Barker S, Friedlander P, Feilding A, Pascual JC, Riba J (2016). Exploring the therapeutic potential of Ayahuasca: acute intake increases mindfulness-related capacities. Psychopharmacology.

[CR38] Trichter S (2010) Ayahuasca beyond the Amazon the benefits and risks of a spreading tradition. J Transpers Psychol 42:131–148

[CR39] Trichter S, Klimo J, Krippner S (2009). Changes in spirituality among ayahuasca ceremony novice participants. J Psychoactive Drugs.

[CR40] Vazquez C, Duque A, Hervas G (2013). Satisfaction with life scale in a representative sample of Spanish adults: validation and normative data. Span J Psychol.

[CR41] Veehof MM, Peter M, Taal E, Westerhof GJ, Bohlmeijer ET (2011). Psychometric properties of the Dutch five facet mindfulness questionnaire (FFMQ) in patients with fibromyalgia. Clin Rheumatol.

[CR42] Viol A, Palhano-Fontes F, Onias H, de Araujo DB, Viswanathan GM (2017). Shannon entropy of brain functional complex networks under the influence of the psychedelic Ayahuasca. Sci Rep.

[CR43] Wardenaar Klaas J., Wanders Rob B. K., Jeronimus Bertus F., de Jonge Peter (2017). The Psychometric Properties of an Internet-Administered Version of the Depression Anxiety and Stress Scales (DASS) in a Sample of Dutch Adults. Journal of Psychopathology and Behavioral Assessment.

